# Partial thrombosis of the corpus cavernosum

**DOI:** 10.1590/0100-3984.2016.0075

**Published:** 2018

**Authors:** Tatiana Bagrichevsky Autran, Alessandro Severo Alves de Melo, Fabio Noro, Bernardo Tessarollo, Márcio Miguez

**Affiliations:** 1 Rede D'Or São Luiz - Hospital Barra D'Or, Rio de Janeiro, RJ, Brazil; 2 Hospital Universitário Antônio Pedro (HUAP), Niterói, RJ, Brazil; 3 Universidade Federal do Rio de Janeiro (UFRJ), Rio de Janeiro, RJ, Brazil; 4 Universidade do Estado do Rio de Janeiro (UERJ), Rio de Janeiro, RJ, Brazil

**Figure 1:**
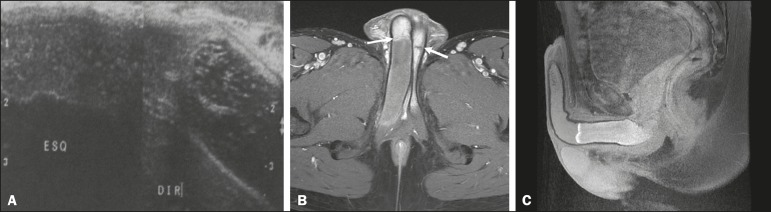
**A:** Comparative, longitudinal ultrasound image of the penis, showing a thrombus in the proximal third of the right corpus cavernosum. **B:** Axial gadolinium contrast-enhanced T1-weighted MRI scan, showing the fibrous septa in both corpora cavernosa (white arrows) and the thrombus in right corpus cavernosum. **C:** Sagittal T1-weighted MRI scan with fat suppression, showing the thrombus in the proximal third of the right corpus cavernosum.

Dear Editor,

A 24-year-old, previously healthy male presented with sudden onset pain at the base of the penis, radiating to the scrotum. Color Doppler ultrasound revealed a thrombus, measuring 9 cm on its longest (longitudinal) axis, in the proximal third of the right corpus cavernosum (Figure 1A). Magnetic resonance imaging (MRI) of the penis confirmed the thrombosis (Figures 1B and 1C) and revealed, at the junction of the crus and the corpus cavernosum (Figure 1B), a thin membrane that divided the thrombus-free part from the part with thrombosis.

Partial thrombosis of the corpus cavernosum is an extremely rare condition, only 56 cases having been reported worldwide by 2015. It occurs in the proximal segment of the corpus cavernosum, usually in men, the mean age of affected individuals being 32 years^1^, and is also known as partial priapism^2^. Most cases appear to be related to perineal compression. The main complication is erectile dysfunction. Of the cases reported in the literature, 59% were in cyclists. The thrombosis is unilateral in 75% of cases and bilateral in 25%.

The pathophysiology of partial thrombosis of the corpus cavernosum is related to the fibrous septum and to the venous system of the proximal third of the penis, which drains into the cavernous veins and internal pudendal veins, all of which are affected by perineal compression. Because there is a mechanical barrier (the fibrous septum), blood cannot reach the distal two-thirds of the penis, where the dorsal veins handle the drainage. The resulting venous stasis predisposes to thrombus formation^1^.

Ultrasound imaging aspects include increased volume of the affected segment of the corpus cavernosum, with heterogeneous material and without vascularization on color Doppler. On Doppler ultrasound, no blood flow is detected within the thrombus, although a system of collateral vessels can be seen at the periphery. At the subacute stage, approximately four weeks later, collateral vessels are seen growing within the thrombus, probably s an early sign of resolution of thrombosis. MRI shows an increase in the volume of the corpus cavernosum, associated with the thrombus. The aspect of the affected segment depends on the age of the thrombus. In comparison with a normal corpus cavernosum, one containing a thrombus is initially hyperintense in T1-weighted sequences and hypointense in T2-weighted sequences^3^. MRI scans can also show the thin membrane separating the rigid part from the flaccid part, which presents a low-intensity signal in the various sequences^4,5^. The thrombus does not show contrast medium uptake. Because it is a poorly understood condition, partial thrombosis of the corpus cavernosum might be underdiagnosed^6^. Treatment should preferably be conservative, with an antiplatelet agent, an anticoagulant, and analgesics^7^. Surgical treatment should be reserved for cases of refractory pain, impotence, or recurrence^8^.
